# Basal Cell Carcinoma Pathology Requests and Reports Are Lacking Important Information

**DOI:** 10.1155/2019/4876309

**Published:** 2019-01-03

**Authors:** Firas Al-Qarqaz, Khaldon Bodoor, Awad Al-Tarawneh, Haytham Eloqayli, Wisam Al Gargaz, Diala Alshiyab, Jihan Muhaidat, Mohammad Alqudah, Rowida Almomani, Maha Marji

**Affiliations:** ^1^Department of Dermatology, Jordan University of Science and Technology, P.O. Box 3030, Irbid, Jordan; ^2^Department of Applied Biology, Jordan University of Science and Technology, Irbid, Jordan; ^3^Department of Internal Medicine and Forensic Medicine, Faculty of Medicine, Mutah University, Mu'tah, Jordan; ^4^Department of Neurosurgery, Faculty of Medicine, Jordan University of Science and Technology, Irbid, Jordan; ^5^Department of Special Surgery, Jordan University of Science and Technology, Irbid, Jordan; ^6^Department of Pathology, Jordan University of Science and Technology, Irbid, Jordan; ^7^Department of Medical Laboratory Sciences, Jordan University of Science and Technology, Irbid, Jordan

## Abstract

**Introduction:**

Basal cell carcinoma (BCC) is the most common cancer affecting humans. Luckily it has negligible risk for metastasis; however it can be locally destructive to surrounding tissue. The diagnosis of this tumor relies on clinical and dermoscopic features; however confirmation requires biopsy and histologic examination. Based on clinical and pathologic findings, BCC is classified as low or high risk subtype. The clinician requesting pathology examination for BCC should provide the pathologist with detailed information including patient details, relevant clinical and medical history, site and type of the biopsy, and whether this is a primary or recurrent lesion. The pathologist on the other hand should write an adequate report containing a minimum of core set of parameters including type of BCC, depth of invasion, presence of lymphovascular or perineural invasion, and the excision margins.

**Objectives:**

The objective of this study is to evaluate whether requests by clinicians and pathology reports of BCC are adequate.

**Methods:**

This is a retrospective analysis done at the dermatology department, faculty of medicine at Jordan University of Science and Technology, Irbid, Jordan. Reports for the period from January 2003 to December 2017 were retrieved and analyzed for data completeness.

**Results:**

Most clinical request forms of BCC provided by clinicians are inadequate and lack important relevant information especially in regard to lesion history, patient medical history, and whether BCC is a primary or a recurrent one. Pathology reports for BCC cases also have significant deficiency especially in describing the histologic subtype, depth of invasion, and presence of lymphovascular and perineural invasion. However, the tumor excision margins are adequately described in almost all reports.

**Conclusions:**

The study shows that clinicians do not provide adequate clinical information when submitting a request for histopathologic examination of BCC. Similarly, pathologists write incomplete reports that lack important pathologic features. Having pre-set forms (electronic proforma) can help overcome missing information.

## 1. Introduction

BCC is the most common cancer affecting populations with fair skin colors [1, 2]. Although it has almost no tendency for metastasis it still can cause significant local morbidity depending on affected site. There are several guidelines which address the various aspects of management of this common cancer [3–5].

The diagnosis of BCC can be suspected from clinical findings including rolled margins, telangiectasia, and shiny surface along with very slow growth mainly in sun-exposed sites especially in individuals with fair skin color. Additionally, dermoscopic examination can help in diagnosis with identification of one or more of dermoscopic features for BCC [6]. Confirmation of diagnosis requires histopathologic examination of biopsy [7, 8]. When submitting the request for histopathologic examination, the clinician should provide adequate relevant clinical information that will help the pathologist examining the specimen. Such information should include, in addition to patient details, clinical description of the lesion (e.g., duration, growth over time, and ulceration), relevant medical history of the patient (e.g., immune suppression, previous radiotherapy, and burns), biopsy type (e.g., punch, incisional), site of biopsy, and whether this is a primary or recurrent BCC [9–12]. Similarly, the histopathology report should be informative and contain adequate information that can help the clinician identify the group of patients with high risk types of BCC which help clinicians in planning the subsequent management steps. The report should include, in addition to the diagnosis, several additional features including completeness of excision and whether tumor cells are reaching any excision margin, the histologic type (e.g., morpheaform/sclerosing, basosquamous, infiltrative, or micronodular), depth of invasion (invasion beyond reticular dermis is feature of aggressive tumor), and perineural involvement and lymphovascular invasion [13–15]. These histologic features are required for accurate diagnosis of BCC as highlighted by BCC management guidelines [3, 4].

The main objective of this study is to assess the completeness of clinicians request forms and pathology reports for cases of BCC at King Abdullah University Hospital, Irbid, Jordan.

## 2. Materials and Methods

BCC pathology reports issued by the pathology department at the Jordan University of Science and Technology, Irbid, Jordan, between the years 2003 and 2017 were reviewed and data regarding the clinical and histologic description of BCC were extracted from these reports. Clinical data examined include patients demographics, lesion history, medical history, biopsy type, and site. The pathology data analyzed include histological subtypes, depth of invasion, presence of perineural/lymphovascular invasion, and excision margins.

Analysis of results was done using SPSS software version 21 (IBM Corporation, USA). Chi-square test of independence and Mann-Whitney test were used to analyze the relationship between variables. P value significance was determined at 0.05 or less.

Data access is restricted as data is owned by the institution where study was done; however upon request the approvals may be obtained from ethics committee as appropriate.

## 3. Results

There were 335 cases of BCC (214 males and 121 females; 1.76:1). Males were diagnosed at a slightly younger age than females (average age males 61.9 years and females 65.4 years). Dermatologists were the referring specialty in 72 of these cases whereas 263 cases were referred by other specialties.

The request forms for BCC cases provided by the clinicians to the pathologists were deficient and missing important clinical information. The clinical description of lesion was absent from almost three quarters (76.4%) of request forms (Table 1). Medical history and whether lesion was primary or recurrent were absent from most request forms. However, biopsy type was mentioned in around 75% of requests from both dermatologists and other specialties. The request forms included adequate information about site of biopsy and patient details for almost all patients. Requests from dermatologists included more clinical information regarding lesion clinical description and biopsy type when compared to requests from other specialties. Table 1 shows the details of these findings in request forms for BCC patients.

The pathology reports similarly showed deficiency in histologic reporting of BCC. These deficiencies in reporting were evident for most core histopathological parameters including subtype, depth of invasion, and presence of perineural/lymphovascular invasion. However, excision margins were described adequately in the majority of the reports. The histologic subtype of BCC was mentioned in only 119 cases (35.5%) (Table 2).

**Figure 1:**
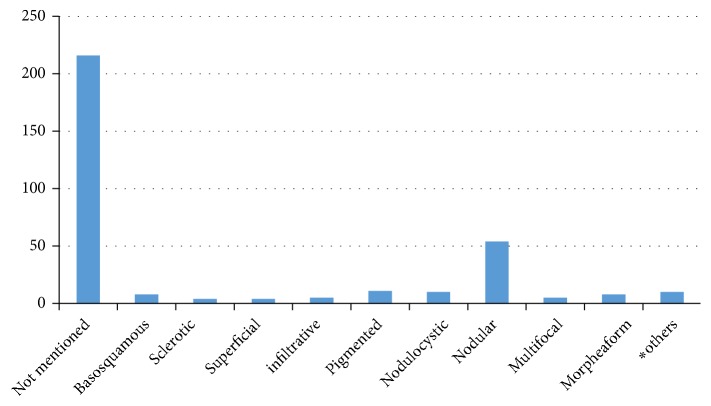
**Subtypes of BCC: terminology and numbers.** *∗*Others: cystic, fibroepithelioma of pinkus, invasive, keratotic, microcystic, micronodular.

An important finding of this study is the inconsistency in the terminology used for describing the various subtypes of BCC. The pathology reports included 15 different terminologies for BCC subtypes. Nodular and nodulocystic BCC were the most common subtypes accounting for around 54% (n=55) of all cases. Other subtypes of BCC described in the reports are shown in Figure 1.

## 4. Discussion

BCC is generally a locally destructive tumor; however the behavior of this tumor is not uniform with patients having either low risk or high risk types. The identification of high risk types is based on patient clinical history, tumor characteristics, and histological features. Patient clinical factors include demographic information (age and sex), immune suppression, organ transplant, prior burn or radiotherapy, and whether this is a primary or recurrent tumor [16]. Tumor-related features include tumor size and location (e.g., nose and periorificial skin denote higher risk areas). Histologic features of high risk subtypes include aggressive histologic subtypes, deeper invasion beyond reticular dermis, and perineural/lymphovascular involvement [15, 17]. It will be interesting to see whether dermoscopic examination features will fit into this assessment. When a clinician is evaluating a patient with BCC, all these factors should be taken into consideration so that risk stratification and treatment are more appropriately planned.

In this study, the data provided by clinicians to the pathologists regarding the clinical information of BCC cases is inadequate. This was evident by the lack of information regarding medical history, lesion history, and whether the tumor is primary or recurrent. In our opinion, this could be due to several factors. For clinicians, pathologic confirmation of diagnosis is the primary concern; however they seem to underestimate or even are unaware of the importance of providing the pathologist with full clinical details. Additionally, absence of a structured request form that requires filling all such information results in variation of information provided. Also, having BCC cases treated by various specialties adds to these discrepancies in request forms.

Histologic evaluation of BCC is of extreme importance not only to establish diagnosis but also to provide predictive information in terms of behavior and risk of recurrence. In the current study, pathology reports were inadequate in providing histological features that are important in determining the behavior of the tumor. For example, pathology reports lacked information about histologic subtype, depth of invasion, and lymphovascular/perineural invasion. These parameters are important in planning appropriate treatment and predicting risk of recurrence and metastasis [18, 19]. However, excision margins were adequately described in almost all pathology reports. The terminology used to describe the different subtypes of BCC was inconsistent in describing the various subtypes of BCC due to the lack of a consensus on using a recognized subtype classification. A recent document by The Royal College of Pathologists of the UK outlined the standards for histological reporting of BCC and the recommended BCC subtypes terminology [20]. Following such a recognized classification should help avoid such inconsistencies in subtype terminology. The missing information from pathology reports could also be due to misconception about the importance of these features in relation to BCC. Having a structured reporting form for BCC that contains all important histopathologic features can reduce such missing information. BCC reporting by dermatopathologists could also help in reducing inadequacies as they are more specialized in reporting skin histopathology including BCC.

The results of this study highlight inadequacy in providing clinical information and histopathological reporting of BCC. These deficiencies have been reported by several other studies. In a study addressing the minimum data set for reporting BCC from the north of England cancer network, there was inadequate histopathological reporting of BCC [21]. In another study, the reporting of BCC by histopathologists showed considerable variation in histopathological reporting [22]. The defect in pathology reporting is not limited to BCC as highlighted by a survey of 73 institutions carried out by the American College of Pathologists [23].

Based on our results, we recommend that a structured pathology request form should be used by clinicians when referring BCC biopsies for pathology examination. This should contain all relevant clinical information for BCC including patient demographics, medical history, lesion history, biopsy site and type, and whether the tumor is primary or recurrent. All clinicians managing BCC patients should be aware of the importance of these factors in confirming the diagnosis by pathologists. Additionally, BCC pathology reports should be designed to include all important histopathologic parameters to provide a complete diagnosis for BCC. Preferably, such report could be an electronic form that avoids the pitfalls of “free text” reporting. Having a dermatopathologist assess BCC cases would also be of importance in terms of accurate BCC diagnosis.

This study highlights inadequate clinical and histopathological reporting of BCC. Having electronic predesigned forms would be very helpful in minimizing missing important clinical and pathological information which could be detrimental to patient outcome.

## Figures and Tables

**Table 1:** Data provided by clinicians in request forms.

Reported data	Dermatologist (N=72)		Other Specialties (N=263)		*p*-value
	Data provided	Data absent	Data provided	Data absent	

Lesion history	34 (47%)	38 (53%)	45 (17.1%)	218 (82.9%)	<0.001

Medical history	1 (1.4%)	71 (98.6%)	1 (0.4%)	262 (99.6%)	0.3

Primary vs recurrent	11 (15.3%)	61 (84.7%)	35 (13.3%)	228 (86.7%)	0.6

Biopsy type	60 (83 %)	12 (17%)	194 (73.4%)	70 (26.6%)	0.001

Biopsy site	66 (91. 5%)	6 (8.5%)	255 (96.6%)	9 (3.4%)	0.1

**Table 2:** Histopathologic features in reports of BCC.

Histopathologic Feature	Adequately described Number (%)	Absent from report Number (%)
Histologic subtype	119 (35.5)	216 (64.5%)

Depth of invasion	55 ( 16.4)	280 (83.6 )

Excision margins	334 ( 99.7)	1 (0.3 )

Lymphovascular invasion	39 (11.6 )	296 ( 88.4)

Perineural invasion	44 (13.1 )	291 ( 86.9)

## Data Availability

The data used to support the findings of this study are available from the corresponding author upon request.
