# Erratum to “Basal Cell Carcinoma Pathology Requests and Reports Are Lacking Important Information”

**DOI:** 10.1155/2019/6846428

**Published:** 2019-07-14

**Authors:** Firas Al-Qarqaz, Khaldon Bodoor, Awad Al-Tarawneh, Haytham Eloqayli, Wisam Al Gargaz, Diala Alshiyab, Jihan Muhaidat, Mohammad Alqudah, Rowida Almomani, Maha Marji

**Affiliations:** ^1^Department of Dermatology, Jordan University of Science and Technology, P.O. Box 3030, Irbid, Jordan; ^2^Department of Applied Biology, Jordan University of Science and Technology, Irbid, Jordan; ^3^Department of Internal Medicine and Forensic Medicine, Faculty of Medicine, Mutah University, Mu'tah, Jordan; ^4^Department of Neurosurgery, Faculty of Medicine, Jordan University of Science and Technology, Irbid, Jordan; ^5^Department of Special Surgery, Jordan University of Science and Technology, Irbid, Jordan; ^6^Department of Pathology, Jordan University of Science and Technology, Irbid, Jordan; ^7^Department of Medical Laboratory Sciences, Jordan University of Science and Technology, Irbid, Jordan

In the article titled “Basal Cell Carcinoma Pathology Requests and Reports Are Lacking Important Information” [1], an Acknowledgments section should be added as follows:
